# Clustering of Five Health-Related Behaviors for Chronic Disease Prevention Among Adults, United States, 2013

**DOI:** 10.5888/pcd13.160054

**Published:** 2016-05-26

**Authors:** Yong Liu, Janet B. Croft, Anne G. Wheaton, Dafna Kanny, Timothy J. Cunningham, Hua Lu, Stephen Onufrak, Ann M. Malarcher, Kurt J. Greenlund, Wayne H. Giles

**Affiliations:** Author Affiliations: Janet B. Croft, Anne G. Wheaton, Dafna Kanny, Timothy J. Cunningham, Hua Lu, Stephen Onufrak, Ann M. Malarcher, Kurt J. Greenlund, Wayne H. Giles, National Center for Chronic Disease Prevention and Health Promotion, Centers for Disease Control and Prevention, Atlanta, Georgia.

## Abstract

**Introduction:**

Five key health-related behaviors for chronic disease prevention are never smoking, getting regular physical activity, consuming no alcohol or only moderate amounts, maintaining a normal body weight, and obtaining daily sufficient sleep. The objective of this study was to estimate the clustering of these 5 health-related behaviors among adults aged 21 years or older in each state and the District of Columbia and to assess geographic variation in clustering.

**Methods:**

We used data from the 2013 Behavioral Risk Factor Surveillance System (BRFSS) to assess the clustering of the 5 behaviors among 395,343 BRFSS respondents aged 21 years or older. The 5 behaviors were defined as currently not smoking cigarettes, meeting the aerobic physical activity recommendation, consuming no alcohol or only moderate amounts, maintaining a normal body mass index (BMI), and sleeping at least 7 hours per 24-hour period. Prevalence of having 4 or 5 of these behaviors, by state, was also examined.

**Results:**

Among US adults, 81.6% were current nonsmokers, 63.9% obtained 7 hours or more sleep per day, 63.1% reported moderate or no alcohol consumption, 50.4% met physical activity recommendations, and 32.5% had a normal BMI. Only 1.4% of respondents engaged in none of the 5 behaviors; 8.4%, 1 behavior; 24.3%, 2 behaviors; 35.4%, 3 behaviors; and 24.3%, 4 behaviors; only 6.3% reported engaging in all 5 behaviors. The highest prevalence of engaging in 4 or 5 behaviors was clustered in the Pacific and Rocky Mountain states. Lowest prevalence was in the southern states and along the Ohio River.

**Conclusion:**

Additional efforts are needed to increase the proportion of the population that engages in all 5 health-related behaviors and to eliminate geographic variation. Collaborative efforts in health care systems, communities, work sites, and schools can promote all 5 behaviors and produce population-wide changes, especially among the socioeconomically disadvantaged.

## Introduction

In 1982, the Alameda County (California) Study identified 5 key lifestyle behaviors for preventing chronic disease as being significantly associated with reduced mortality (1). The behaviors were never smoking, getting regular physical activity, drinking fewer than 5 drinks at one sitting, maintaining a normal body weight, and sleeping 7 to 8 hours per night. In 1985, the first assessment of clustering of these 5 health-related behaviors and 2 additional eating behaviors (not skipping breakfast and not snacking between meals) in the US population demonstrated disparities among racial/ethnic and socioeconomic groups (2).

Since 1985, most studies that have assessed clustering of health behaviors related to chronic disease or their impact on mortality (3–7) have not included getting sufficient sleep. The primary reason for this omission is the lack of national surveillance data on sleep duration until 2004 and lack of a formal recommendation for hours of sleep for adults until 2015 (8). In 2015, the American Academy of Sleep Medicine and Sleep Research Society recommended that adults should regularly get 7 or more hours of sleep nightly (8). One study found that US adults who slept 7 to 8 hours daily reported lower prevalence of cigarette smoking, drinking 5 or more servings of alcohol beverages in one day during the past year, physical inactivity, and obesity than adults who slept less than 7 hours (9). However, insufficient sleep is an independent risk factor for mortality and the development of chronic diseases after accounting for other health-related behaviors (10).

No current estimates exist of the clustering of all 5 health-related behaviors among US adults, although one recent study examined geographic variation by state of getting sufficient sleep (11). The objective of this study was to estimate the clustering of these 5 health-related behaviors among adults aged 21 years or older in 50 states and the District of Columbia and to assess geographic and sociodemographic variations in clustering.

## Methods

### Data sources

We used data from the 2013 Behavioral Risk Factor Surveillance System (BRFSS), an ongoing random-digit–dialed telephone survey of noninstitutionalized US adults aged 18 years or older that is conducted by state health departments with assistance from the Centers for Disease Control and Prevention. The survey sample is selected through a multistage sampling design of households with either landline or cellular telephones. Response rates for BRFSS are calculated using standards set by the American Association of Public Opinion Research Response Rate, Formula 4 (www.aapor.org/AAPOR_Main/media/publications/Standard-Definitions20169theditionfinal.pdf). The response rate is the number of respondents who completed the survey as a proportion of all eligible and likely eligible persons. The median survey response rate for all 50 states and the District of Columbia in 2013 was 45.9% and ranged from 29.0% in Alabama to 59.2% in North Dakota (www.cdc.gov/brfss/annual_data/2013/pdf/2013_DQR.pdf). Additional information about the 2013 BRFSS is available (www.cdc.gov/brfss/annual_data/annual_2013.html).

All 5 health-related behaviors assessed in this study are included as topics in Healthy People 2020. A normal body weight is defined as a body mass index (BMI, in kg/m^2^), based on self-reported height and weight, of 18.5 to 24.9. Nondrinkers or moderate drinkers are people who drank no alcohol or drank alcohol in moderation during the past 30 days. Moderate drinking was defined as drinking up to 2 alcoholic drinks per day for men and up to 1 drink per day for women (12), no reported binge drinking (5 or more drinks on one occasion for men and 4 or more drinks for women), and no heavy drinking (15 or more drinks per week for men and 8 or more drinks per week for women during the past 30 days) (12). Engaging in aerobic physical activity was defined as self-reported aerobic physical activity in the past month of at least 150 minutes per week of moderate-intensity physical activity, 75 minutes per week of vigorous-intensity physical activity, or a combination of moderate and vigorous activity. Current nonsmoking was defined as respondents self-reporting not smoking 100 cigarettes or more during their lifetime or having smoked at least 100 cigarettes during their lifetime but not smoking at the time of the survey. Sufficient sleep was defined as reporting usually getting 7 or more hours of sleep during a 24-hour period (10).

The study population was restricted to adults of legal drinking age (≥21 y) who resided in one of the 50 states or the District of Columbia, excluding pregnant women and respondents who had a missing value for age or any of the 5 health-related behaviors. The final study population was 395,343 adults aged 21 years or older.

### Statistical analysis

Age-adjusted weighted prevalence with 95% confidence intervals (CIs) for the number (0–5) of health-related behaviors was calculated by state and by selected demographic characteristics by using SAS-callable SUDAAN, version 10.0.1 (Research Triangle Institute). Calculations accounted for the complex sampling design. All significant differences (*P* < .05) between subgroups were determined by *t* tests.

## Results

The highest crude percentage of individual health-related behaviors was current nonsmoking (81.6%), followed by sufficient sleep (63.9%), nondrinking or moderate drinking (63.1%), meeting recommendations for aerobic physical activity (50.4%), and maintaining a normal BMI (32.5%). A small proportion of respondents (1.4%) reported none of the 5 health-related behaviors; 8.4% reported one behavior, 24.3% reported 2 behaviors, 35.4% reported 3 behaviors, 24.3% reported 4 behaviors, and only 6.3% reported all 5 behaviors (Table 1). Adults aged 65 years or older had the highest percentage (10.1%) of all 5 health-related behaviors compared with other age groups. A higher age-adjusted percentage of all 5 health-related behaviors was observed among women (7.6%) than men (4.8%) and among Asian respondents (11.6%) than all other racial/ethnic groups (*P* < .001 for both comparisons). Compared with non-Hispanic white respondents, non-Hispanic blacks, Hispanics, and American Indians/Alaska Natives (*P* ≤ .001 for all comparisons) had lower age-adjusted percentages of all 5 health-related behaviors. The age-adjusted percentage of reporting all 5 health-related behaviors increased with greater educational attainment.

**Table 1:** Age-Specific and Age-Adjusted Percentage^a^ of Adults Aged ≥21 Years Reporting Health-Related Behaviors, by Selected Characteristics, Behavioral Risk Factor Surveillance System, United States, 2013

Characteristic	No. of Survey Respondents^b^	Weighted % (95% CI), by No. of Behaviors Reported					
		0	1	2	3	4	5
**Total, crude**	395,343	1.4 (1.3–1.5)	8.4 (8.2–8.6)	24.3 (24.0–24.6)	35.4 (35.1–35.7)	24.3 (24.0–24.5)	6.3 (6.1–6.4)
**Total, age–adjusted**	395,343	1.5 (1.4–1.6)	8.7 (8.5–8.9)	24.5 (24.2–24.8)	35.2 (34.9–35.5)	24.0 (23.7–24.3)	6.2 (6.0–6.3)
**Age, y**							
21–24	12,944	2.2 (1.8–2.7)	9.5 (8.7–10.3)	24.2 (23.0–25.4)	32.8 (31.5–34.2)	24.9 (23.7–26.1)	6.4 (5.6–7.1)
25–34	39,546	2.0 (1.8–2.3)	11.2 (10.7–11.7)	27.2 (26.4–28.0)	34.1 (33.3–35.0)	20.3 (19.6–21.0)	5.2 (4.8–5.6)
35–44	49,106	1.8 (1.6–2.0)	10.1 (9.6–10.6)	26.1 (25.3–26.8)	34.9 (34.1–35.7)	21.9 (21.2–22.7)	5.2 (4.8–5.5)
45–54	70,152	1.7 (1.5–1.9)	10.3 (9.8–10.7)	27.2 (26.5–27.9)	34.4 (33.7–35.1)	21.5 (20.9–22.1)	5.0 (4.6–5.3)
55–64	90,411	1.0 (0.8–1.1)	7.8 (7.4–8.1)	24.6 (24.0–25.2)	36.8 (36.1–37.5)	24.3 (23.7–24.9)	5.6 (5.3–5.8)
≥65	133,184	0.2 (0.2–0.3)	2.9 (2.7–3.1)	17.2 (16.7–17.6)	37.5 (36.9–38.0)	32.1 (31.6–32.6)	10.1 (9.8–10.4)
**Sex**							
Men	166,193	1.7 (1.6–1.9)	9.9 (9.6–10.2)	26.3 (25.8–26.7)	35.0 (34.5–35.5)	22.3 (21.9–22.7)	4.8 (4.6–5.0)
Women	229,150	1.2 (1.1–1.3)	7.4 (7.1–7.6)	22.7 (22.3–23.1)	35.4 (35.0–35.9)	25.7 (25.3–26.2)	7.6 (7.3–7.8)
**Race/ethnicity**							
White, non–Hispanic	314,964	1.4 (1.3–1.5)	8.8 (8.5–9.0)	24.2 (23.9–24.5)	34.9 (34.6–35.3)	24.2 (23.9–24.6)	6.4 (6.3–6.6)
Black, non–Hispanic	29,901	2.2 (1.8–2.5)	11.1 (10.4–11.7)	28.0 (27.0–28.9)	35.7 (34.6–36.8)	19.7 (18.8–20.6)	3.5 (3.1–3.8)
Hispanic	22,379	1.3 (1.1–1.6)	7.6 (7.0–8.2)	26.4 (25.3–27.6)	36.4 (35.2–37.6)	23.3 (22.2–24.4)	4.9 (4.4–5.5)
American Indian/Alaska Native	5,990	2.0 (1.2–2.8)	12.1 (10.3–13.9)	27.9 (25.3–30.5)	32.2 (29.6–34.8)	21.4 (18.7–24.1)	4.4 (3.3–5.6)
Asian	6,935	0.6 (0.3–0.9)	4.3 (3.4–5.2)	17.9 (15.7–20.1)	35.3 (32.9–37.6)	30.4 (28.3–32.5)	11.6 (10.0–13.1)
Native Hawaiian/Pacific Islander	654	1.3 (0.3–2.3)^c^	16.5 (9.7–23.3)	22.3 (16.7–27.8)	26.5 (20.5–32.5)	27.7 (20.6–34.9)	5.7 (2.7–8.8)
Multiracial, non–Hispanic	7,243	2.0 (1.2–2.8)	12.0 (10.0–14.1)	25.9 (23.6–28.2)	35.6 (32.9–38.3)	19.4 (17.3–21.5)	5.1 (3.8–6.4)
**Education**							
Less than high school graduate	29,648	2.3 (1.9–2.7)	11.9 (11.2–12.7)	29.1 (28.0–30.2)	34.6 (33.4–35.9)	18.7 (17.7–19.6)	3.4 (2.9–3.8)
High school graduate or GED	110,984	2.1 (1.9–2.2)	10.9 (10.4–11.3)	26.7 (26.1–27.3)	34.5 (33.9–35.1)	21.0 (20.4–21.5)	4.9 (4.6–5.2)
Some college	108,707	1.6 (1.5–1.8)	9.1 (8.8–9.5)	25.3 (24.7–25.8)	35.1 (34.5–35.7)	22.9 (22.4–23.5)	5.9 (5.6–6.2)
College graduate	145,521	0.5 (0.4–0.5)	4.9 (4.7–5.1)	19.6 (19.2–20.0)	35.8 (35.3–36.3)	30.3 (29.8–30.8)	8.9 (8.6–9.1)

Abbreviations: CI, confidence interval, GED, general educational development.

a Age-adjusted to the 2000 projected US population, except for age groups.

b Unweighted sample of respondents. Categories might not sum to survey total because of respondents with missing data on characteristics.

c Estimates are unreliable because relative standard error >0.3 or n < 50.

The age-adjusted percentage for each number of health-related behaviors varied by state (Table 2). Utah had the highest percentage of respondents reporting all 5 health-related behaviors (11.3%), and 7 states (Arkansas, Louisiana, Mississippi, North Dakota, Rhode Island, Tennessee, and Wisconsin) had percentages below 5.0%.

**Table 2:** Age-Adjusted Percentage^a^ of Adults Aged ≥21 Years Reporting Health-Related Behaviors, by State, Behavioral Risk Factor Surveillance System, United States, 2013

State	No.^b^	Weighted % (95% CI), by No. of Behaviors Reported					
		0	1	2	3	4	5
Alabama	5,411	1.0 (0.6–1.4)	8.4 (7.3–9.6)	26.5 (24.5–28.4)	35.5 (33.5–37.5)	23.2 (21.5–24.9)	5.4 (4.5–6.3)
Alaska	3,792	2.0 (1.2–2.8)	9.8 (8.2–11.3)	24.6 (22.6–26.6)	34.0 (31.8–36.1)	23.6 (21.7–25.4)	6.1 (5.0–7.1)
Arizona	3,430	0.8 (0.4–1.1)	8.0 (6.2–9.9)	22.9 (20.1–25.6)	34.8 (31.8–37.8)	26.7 (24.1–29.4)	6.8 (5.5–8.1)
Arkansas	4,273	2.3 (1.5–3.1)	10.6 (9.0–12.2)	27.3 (25.2–29.5)	34.7 (32.5–36.9)	20.9 (19.1–22.7)	4.2 (3.4–4.9)
California	8,893	0.9 (0.6–1.1)	6.3 (5.6–7.1)	21.5 (20.2–22.8)	35.5 (34.1–37.0)	27.8 (26.5–29.1)	7.9 (7.1–8.7)
Colorado	11,043	1.1 (0.8–1.5)	6.6 (5.9–7.3)	19.5 (18.4–20.5)	34.6 (33.4–35.8)	29.8 (28.7–30.9)	8.4 (7.8–9.1)
Connecticut	6,351	1.7 (1.1–2.3)	9.5 (8.3–10.7)	24.7 (23.0–26.4)	33.7 (31.9–35.6)	23.8 (22.2–25.4)	6.5 (5.6–7.4)
Delaware	4,327	1.5 (1.0–2.0)	8.6 (7.3–9.9)	27.4 (25.3–29.4)	35.5 (33.4–37.6)	21.5 (19.7–23.2)	5.6 (4.5–6.7)
District of Columbia	3,927	1.9 (1.0–2.9)	7.9 (6.5–9.4)	20.7 (18.7–22.7)	37.0 (34.6–39.4)	26.2 (24.1–28.3)	6.2 (5.2–7.2)
Florida	26,727	1.1 (0.8–1.3)	8.3 (7.4–9.2)	25.2 (23.8–26.6)	36.4 (34.9–38.0)	23.4 (22.1–24.7)	5.5 (4.8–6.3)
Georgia	6,488	1.5 (1.0–1.9)	8.9 (7.9–10.0)	24.1 (22.6–25.6)	35.0 (33.4–36.7)	24.4 (23.0–25.9)	6.0 (5.3–6.7)
Hawaii	6,775	1.0 (0.6–1.5)	6.3 (5.3–7.2)	21.3 (19.7–22.8)	35.1 (33.3–36.8)	27.1 (25.5–28.7)	9.2 (8.2–10.3)
Idaho	4,651	2.3 (1.3–3.3)	7.3 (6.1–8.5)	20.3 (18.6–22.1)	35.5 (33.4–37.7)	25.9 (24.1–27.7)	8.6 (7.4–9.9)
Illinois	4,981	1.4 (0.9–1.9)	8.2 (7.0–9.4)	26.1 (24.2–28.0)	34.8 (32.8–36.8)	23.5 (21.7–25.2)	6.0 (5.1–7.0)
Indiana	8,357	1.5 (1.1–1.9)	11.1 (10.1–12.2)	26.2 (24.8–27.6)	35.0 (33.6–36.5)	21.1 (19.9–22.3)	5.0 (4.5–5.5)
Iowa	6,845	1.8 (1.2–2.3)	10.8 (9.6–12.0)	25.0 (23.5–26.5)	34.6 (33.0–36.2)	22.4 (21.0–23.7)	5.5 (4.8–6.1)
Kansas	19,438	1.3 (1.0–1.5)	8.2 (7.7–8.8)	23.5 (22.7–24.3)	35.4 (34.5–36.3)	25.4 (24.6–26.2)	6.2 (5.8–6.6)
Kentucky	8,967	1.5 (1.1–2.0)	10.7 (9.6–11.9)	27.1 (25.6–28.7)	34.9 (33.3–36.6)	20.6 (19.3–21.9)	5.0 (4.3–5.7)
Louisiana	4,332	2.0 (1.1–2.9)	9.9 (8.3–11.6)	27.4 (25.0–29.8)	34.8 (32.5–37.2)	21.0 (19.0–23.0)	4.8 (3.9–5.8)
Maine	6,922	1.6 (1.0–2.2)	8.3 (7.3–9.4)	26.2 (24.6–27.8)	34.0 (32.3–35.6)	23.7 (22.3–25.1)	6.2 (5.4–6.9)
Maryland	10,145	1.2 (0.8–1.5)	8.9 (7.9–10.0)	25.7 (24.2–27.1)	35.0 (33.5–36.5)	23.3 (22.0–24.7)	5.9 (5.1–6.6)
Massachusetts	11,900	1.3 (0.9–1.8)	8.0 (7.1–8.8)	24.6 (23.2–25.9)	34.8 (33.4–36.2)	24.3 (23.1–25.5)	7.0 (6.4–7.7)
Michigan	10,887	2.0 (1.6–2.4)	9.8 (8.8–10.7)	27.0 (25.7–28.3)	33.9 (32.6–35.2)	21.8 (20.6–22.9)	5.6 (5.0–6.2)
Minnesota	11,827	1.7 (1.2–2.2)	9.4 (8.4–10.4)	24.3 (22.8–25.8)	33.9 (32.3–35.5)	23.8 (22.4–25.3)	6.9 (6.0–7.7)
Mississippi	6,025	1.6 (1.0–2.1)	10.7 (9.4–12.0)	27.4 (25.6–29.2)	34.6 (32.8–36.4)	21.5 (19.9–23.1)	4.3 (3.4–5.1)
Missouri	5,999	1.9 (1.0–2.8)	8.5 (7.4–9.7)	23.8 (22.1–25.6)	36.3 (34.4–38.3)	23.6 (22.0–25.2)	5.8 (5.0–6.7)
Montana	8,317	1.0 (0.7–1.3)	8.7 (7.7–9.6)	22.3 (21.0–23.6)	34.3 (32.7–35.8)	26.5 (25.0–27.9)	7.3 (6.5–8.1)
Nebraska	14,585	1.8 (1.3–2.2)	9.9 (8.9–10.8)	24.3 (23.1–25.5)	36.0 (34.6–37.4)	22.6 (21.4–23.7)	5.5 (4.9–6.0)
Nevada	4,250	1.3 (0.8–1.9)	7.8 (6.3–9.3)	24.8 (22.3–27.3)	35.9 (33.0–38.8)	24.5 (22.0–26.9)	5.8 (4.6–6.9)
New Hampshire	5,314	1.4 (0.9–1.9)	7.8 (6.7–8.9)	23.4 (21.7–25.2)	36.3 (34.4–38.3)	24.2 (22.6–25.8)	6.8 (5.8–7.8)
New Jersey	10,313	1.4 (1.0–1.8)	8.7 (7.8–9.6)	24.7 (23.4–26.0)	36.5 (35.0–38.0)	23.0 (21.7–24.3)	5.7 (5.0–6.3)
New Mexico	7,625	1.3 (0.9–1.7)	7.0 (6.1–7.8)	22.4 (20.9–23.9)	33.6 (31.9–35.2)	27.7 (26.1–29.3)	8.1 (7.2–9.0)
New York	6,991	1.6 (1.1–2.1)	8.7 (7.7–9.7)	26.2 (24.8–27.7)	35.3 (33.8–36.9)	22.4 (21.1–23.6)	5.7 (5.0–6.4)
North Carolina	7,143	1.2 (0.8–1.5)	8.7 (7.7–9.7)	24.3 (22.9–25.8)	36.4 (34.8–38.0)	22.8 (21.5–24.2)	6.6 (5.8–7.3)
North Dakota	6,479	1.7 (1.2–2.2)	11.2 (10.0–12.5)	27.5 (25.8–29.1)	34.9 (33.2–36.6)	20.6 (19.2–22.0)	4.2 (3.5–4.8)
Ohio	9,797	2.3 (1.7–2.9)	10.4 (9.4–11.3)	27.3 (25.9–28.7)	33.4 (31.9–34.9)	21.1 (19.9–22.3)	5.5 (4.8–6.2)
Oklahoma	7,025	1.8 (1.3–2.3)	10.1 (9.1–11.2)	25.1 (23.6–26.5)	34.9 (33.4–36.4)	22.7 (21.4–24.0)	5.4 (4.8–6.1)
Oregon	4,744	1.0 (0.5–1.5)	6.3 (5.2–7.5)	20.2 (18.6–21.9)	34.2 (32.3–36.1)	29.2 (27.4–31.0)	9.0 (7.9–10.1)
Pennsylvania	9,182	2.5 (1.9–3.0)	10.9 (9.9–11.9)	24.8 (23.5–26.1)	34.1 (32.7–35.5)	22.3 (21.1–23.6)	5.4 (4.8–6.1)
Rhode Island	5,226	1.7 (1.1–2.3)	10.5 (9.2–11.8)	26.6 (24.8–28.3)	34.9 (33.0–36.8)	21.7 (20.1–23.3)	4.7 (4.0–5.4)
South Carolina	8,788	1.8 (1.3–2.2)	10.0 (8.9–11.0)	24.7 (23.3–26.1)	36.3 (34.7–37.8)	22.2 (20.9–23.5)	5.1 (4.4–5.7)
South Dakota	5,842	1.6 (0.9–2.2)	8.3 (6.9–9.6)	22.9 (21.0–24.9)	35.5 (33.3–37.8)	26.1 (24.2–28.1)	5.6 (4.6–6.5)
Tennessee	4,510	1.5 (0.9–2.0)	10.5 (9.1–12.0)	26.2 (24.2–28.2)	36.2 (34.1–38.3)	21.2 (19.5–23.0)	4.3 (3.6–5.1)
Texas	8,459	1.8 (1.3–2.2)	8.6 (7.7–9.6)	24.4 (22.9–25.9)	36.2 (34.6–37.9)	23.4 (21.9–24.8)	5.6 (4.8–6.4)
Utah	10,532	0.7 (0.4–0.9)	4.8 (4.3–5.4)	17.5 (16.6–18.5)	33.7 (32.5–34.8)	32.0 (30.9–33.1)	11.3 (10.6–12.0)
Vermont	5,406	0.8 (0.5–1.2)	7.7 (6.6–8.8)	22.3 (20.7–24.0)	35.2 (33.4–37.0)	26.1 (24.5–27.7)	7.9 (7.0–8.8)
Virginia	6,851	1.4 (1.0–1.8)	8.9 (7.9–9.9)	24.7 (23.3–26.2)	35.0 (33.4–36.6)	23.7 (22.4–25.1)	6.2 (5.5–7.0)
Washington	9,518	1.1 (0.8–1.4)	7.9 (7.0–8.7)	22.3 (21.1–23.5)	34.6 (33.3–36.0)	26.5 (25.3–27.7)	7.5 (6.8–8.3)
West Virginia	5,106	1.9 (1.3–2.5)	10.6 (9.4–11.8)	25.2 (23.7–26.8)	35.5 (33.8–37.2)	21.5 (20.1–22.9)	5.3 (4.5–6.0)
Wisconsin	5,259	1.3 (0.8–1.7)	10.0 (8.6–11.5)	25.2 (23.3–27.2)	35.3 (33.1–37.5)	23.7 (21.8–25.6)	4.4 (3.6–5.3)
Wyoming	5,368	1.1 (0.6–1.6)	7.7 (6.6–8.9)	24.1 (22.2–26.0)	33.0 (31.1–34.9)	26.4 (24.7–28.1)	7.6 (6.7–8.6)
Total U.S.	395,343	1.5 (1.4–1.6)	8.7 (8.5–8.9)	24.5 (24.2–24.8)	35.2 (34.9–35.5)	24.0 (23.7–24.3)	6.2 (6.0–6.3)
U.S. median	NA	1.5	8.7	24.7	35.0	23.6	5.8

Abbreviations: CI, confidence interval; NA, not applicable.

a Age–adjusted to the 2000 projected US population.

b Unweighted sample of respondents.

**Figure 1:**
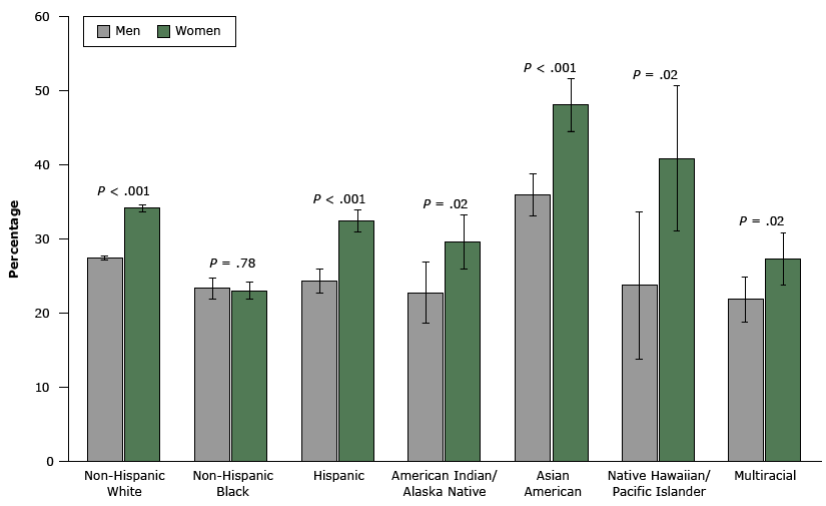
Age-adjusted prevalence of engaging in 4 or 5 health-related behaviors among adults aged 21 years or older, Behavioral Risk Factor Surveillance System, 2013. Error bars indicate 95% confidence intervals. **Table:** 

**Figure 2:**
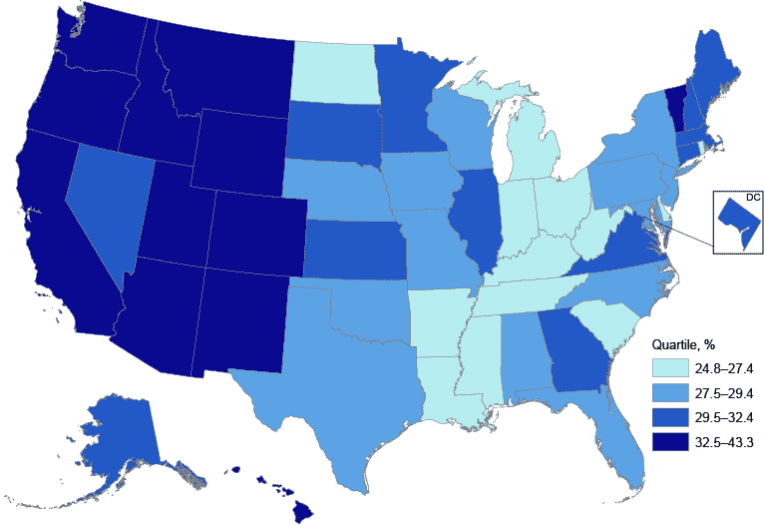
Age-adjusted prevalence of adults aged 21 years or older self-reporting 4 or 5 health-related behaviors, by state and quartile, Behavioral Risk Factor Surveillance System, 2013. **Table:** 

Because a small proportion of respondents reported all 5 health-related behaviors, we compared those reporting 4 or 5 health-related behaviors (30.2% age-adjusted overall) by sex and racial/ethnic group (Figure 1) and by state (Figure 2). A significantly higher percentage of women reported 4 or 5 health-related behaviors than men in all racial/ethnic groups except for non-Hispanic blacks (Figure 1). Lower age-adjusted percentages of respondents reporting 4 or 5 health-related behaviors clustered in many southern states and among states along the Ohio River, whereas higher percentages clustered in the Pacific and Rocky Mountain states (Figure 2).

Race/Ethnicity	Men, % (95% Confidence Interval)	Women, % (95% Confidence Interval)	P Value
Non-Hispanic white	27.5 (27.1–27.9)	34.1 (33.6–34.6)	<.001
Non-Hispanic black	23.3 (21.8–24.8)	23.0 (21.8–24.2)	.78
Hispanic	24.3 (22.6–26.0)	32.4 (30.8–34.0)	<.001
American Indian/Alaska Native	22.7 (18.5–26.9)	29.6 (25.9–33.3)	.02
Asian American	35.9 (33.0–38.8)	48.0 (44.4–51.6)	<.001
Native Hawaiian/Pacific Islander	23.7 (13.7–33.7)	40.8 (31.0–50.6)	.02
Multiracial	21.8 (18.8–24.8)	27.3 (23.8–30.8)	.02

State	Reporting 4 or 5 Health-Related Behaviors, %	Quartile, %
United States, total	30.2	29.5–32.4
Alabama	28.6	27.5–29.4
Alaska	29.7	29.5–32.4
Arizona	33.5	32.5–43.3
Arkansas	25.1	24.8–27.4
California	35.7	32.5–43.3
Colorado	38.2	32.5–43.3
Connecticut	30.3	29.5–32.4
Delaware	27.1	24.8–27.4
District of Columbia	32.4	29.5–32.4
Florida	28.9	27.5–29.4
Georgia	30.4	29.5–32.4
Hawaii	36.3	32.5–43.3
Idaho	34.5	32.5–43.3
Illinois	29.5	29.5–32.4
Indiana	26.1	24.8–27.4
Iowa	27.9	27.5–29.4
Kansas	31.6	29.5–32.4
Kentucky	25.6	24.8–27.4
Louisiana	25.8	24.8–27.4
Maine	29.9	29.5–32.4
Maryland	29.2	27.5–29.4
Massachusetts	31.3	29.5–32.4
Michigan	27.4	24.8–27.4
Minnesota	30.7	29.5–32.4
Mississippi	25.8	24.8–27.4
Missouri	29.4	27.5–29.4
Montana	33.8	32.5–43.3
Nebraska	28.1	27.5–29.4
Nevada	30.3	29.5–32.4
New Hampshire	31.0	29.5–32.4
New Jersey	28.7	27.5–29.4
New Mexico	35.8	32.5–43.3
New York	28.1	27.5–29.4
North Carolina	29.4	27.5–29.4
North Dakota	24.8	24.8–27.4
Ohio	26.6	24.8–27.4
Oklahoma	28.1	27.5–29.4
Oregon	38.2	32.5–43.3
Pennsylvania	27.7	27.5–29.4
Rhode Island	26.4	24.8–27.4
South Carolina	27.3	24.8–27.4
South Dakota	31.7	29.5–32.4
Tennessee	25.5	24.8–27.4
Texas	29.0	27.5–29.4
Utah	43.3	32.5–43.3
Vermont	34.0	32.5–43.3
Virginia	29.9	29.5–32.4
Washington	34.0	32.5–43.3
West Virginia	26.8	24.8–27.4
Wisconsin	28.1	27.5–29.4
Wyoming	34.0	32.5–43.3

## Discussion

This study is the first to describe distributions of the 5 health-related behaviors, including sufficient sleep, related to Healthy People 2020 objectives since the Alameda County Study of 1982, which demonstrated lower mortality risk among adults with these behaviors and disparities among racial/ethnic and socioeconomic groups (2). Our results confirmed that these disparities persisted after 3 decades. Women, older respondents (≥65 y), college graduates, and Asians were more likely to engage in 5 health-related behaviors than men, and other age, education, and racial/ethnic groups. We also found that most US adults did not meet recommendations for aerobic physical activity or did not have a normal BMI. Overall, only 6.3% of the adult population reported engaging in all 5 health-related behaviors in 2013. Although state variations in prevalence of smoking, obesity, physical inactivity, and binge drinking or heavy drinking were mapped separately (www.cdc.gov/brfss/), this is the first geographic assessment of the clustering of these health-related behaviors. This study demonstrates a higher percentage of 5 health-related behaviors in the Pacific and Rocky Mountain states than in southern states.

The lower percentage of people engaging in 4 or more health-related behaviors in the states bordering the Ohio River and southern states compared with other states is consistent with, and indirectly reflects, geographic variations in mortality rates and Medicare hospitalization rates for heart disease and stroke (www.cdc.gov/dhdsp/maps/national_maps) in these states and a high prevalence of chronic obstructive pulmonary disease and diabetes (13–16). Geographic variation and racial/ethnic disparity among respondents reporting 4 or more health-related behaviors reinforce the need for integrated, comprehensive strategies that address all of these behaviors to create sustained population-wide changes; special attention is needed for socioeconomically disadvantaged populations (17).

Our study has several limitations. First, all health-related behaviors are self-reported and subject to reporting bias. Second, BRFSS is a household telephone survey that does not include people living in institutions, long-term care facilities, and prisons; therefore, our results cannot be extrapolated to those groups. Third, because state response rates were relatively low, nonresponse to the telephone survey may have affected results. Fourth, moderate alcohol use was included as a health-related behavior to help reduce the risk of alcohol-attributable harms among current adult drinkers (12), and not to promote the potential health benefit of moderate drinking, which emerging scientific evidence may not support (18). The US Dietary Guidelines also does not recommend that individuals who do not drink alcohol start drinking for any reason (12). Finally, only respondents who provided responses to questions about all 5 health-related behaviors were included in the study. Rates of exclusion from the study were higher among young adults, Hispanics, Native Hawaiians/Pacific Islanders, women, and adults with less education than a high school diploma; these exclusions could have resulted in underestimates or overestimates of health-related behaviors in those groups. However, state variation in exclusions did not follow any pattern that would appear to affect the geographic clustering of health-related behaviors.

Although each health-related behavior has a Healthy People 2020 objective, the 5 behaviors we examined are probably not equal in their health consequences or their amenability to intervention. Therefore, multiple strategies should be employed for the 5 behaviors examined through various avenues that focus on changes in individual behavior and on environmental, policy, and systems changes; we provide examples of such strategies (19–29) (Table 3). First, these strategy examples require linkages with partners in disciplines other than public health as well as stronger linkages between public health and health care than currently exist. Second, although we did not examine children and adolescents in this analysis, health-related behaviors begin in childhood and may be associated with a lower risk of chronic disease in adulthood (30); therefore, strategies to promote health-related behaviors among adults must also include attention to strategies that address children, such as those in early care and education and in school settings. Third, evidence-based environmental approaches in communities, work sites, and schools include providing access to facilities and opportunities to promote lifestyle changes and promoting policies that support and reinforce health practices. In addition, health care system policies can ensure that health care providers assess these key behaviors in office visits and counsel all patients to practice all 5 health-related behaviors. Finally, community–clinical linkages may involve health care providers referring patients with chronic diseases to community-based lifestyle intervention programs and including risk factor reduction via these 5 health-related behaviors in chronic disease self-management programs. These efforts in both the public health sector and health care systems could place a greater emphasis on the promotion of health-related behaviors that prevent chronic diseases and have the potential to support an improved quality of life, reduce development of chronic conditions, reduce demand on the health care system, and improve overall population health (17).

**Table 3:** Examples of Strategies to Support Selected Healthy People 2020 Objectives

Healthy People 2020 Objective	Epidemiology and Surveillance	Environmental Approaches	Health System Strategies	Community–Clinical Linkages
	Collect, analyze, and share data to help identify and solve problems, evaluate public health efforts, and guide and monitor programs and interventions, research, and policies to improve public health.	Promote health and support and reinforce healthy behaviors in schools and child care settings, work sites, and communities.	Improve the delivery and use of clinical and other preventive services that are designed to prevent disease or detect it early, reduce risk factors, and manage complications.	Link community and clinical services to ensure that people with or at high risk of chronic diseases have access to the resources they need to prevent or manage these diseases.
Tobacco Use–1.1: Reduce cigarette smoking among adults	Conduct routine surveillance in BRFSS (adults), YRBSS (adolescents), NYTS (youth), NATS (adults), and YTS (youth) (http://www.cdc.gov/tobacco/data_statistics/index.htm).	Implement comprehensive tobacco control programs (19). Support increases in the price of tobacco products (19). Conduct mass-reach tobacco counter-marketing campaigns (19). Support tobacco-free policies in schools, work sites, public places, multiunit housing, and health care settings (www.thecommunityguide.org/tobacco). Support community mobilization with additional interventions to restrict minors’ access to tobacco products (19). Support state quitline capacity (19).	Expand insurance coverage so that all evidence-based cessation treatments are covered with no barriers to accessing coverage (19). Inform tobacco users and their health care providers of their comprehensive cessation treatment insurance benefits (19). Promote health systems changes including electronic health records with provider reminder systems that integrate tobacco screening and interventions into routine clinical care (19). Promote screening for tobacco use and tobacco cessation treatments (counseling and medication) (19).	Link health care systems with tobacco quitlines and other community-based cessation programs in the state through electronic health records (19). Promote tobacco cessation in chronic disease self-management programs (19).
Physical Activity–2.1: Increase the proportion of adults engaged in aerobic physical activity of at least a moderate intensity for at least 150 min/week or 75 min/week of vigorous intensity, or an equivalent combination	Conduct routine surveillance in BRFSS (adults) and YRBSS (adolescents); SHPPS and Profiles (www.cdc.gov/healthyyouth/data/index.htm).	Promote adoption of physical education/physical activity in schools (20). Promote adoption of physical activity in child care programs and work sites (21). Promote physical activity access and outreach (20). Design streets and communities for safe physical activity (20).	Promote physical activity as a vital sign (www.uspreventiveservicestaskforce.org/Page/Document/RecommendationStatementFinal/healthy-diet-and-physical-activity-counseling-adults-with-high-risk-of-cvd).	Promote physical activity in chronic disease self-management programs (22).
Substance Abuse–15: Reduce the proportion of adults who drank excessively in the previous 30 days	Conduct routine surveillance in BRFSS (adults) and YRBSS (adolescents).	Support Community Guide–recommended strategies (23), such as alcohol-pricing strategies, regulating alcohol density, dram shop liability, and preventing illegal sales.	Promote alcohol screening and brief interventions for adults during routine medical visits (24).	Promote adherence to the Dietary Guidelines on alcohol in chronic disease self-management programs (12).
Nutrition and Weight Status–8: Increase the proportion of adults who are at a healthy weight (18.5–24.9 kg/m^2^)	Conduct routine surveillance in BRFSS (adults) and YRBSS (adolescents).	Promote adoption of food service guidelines/nutrition standards (including competitive foods) in schools, childcare programs, and work sites (20,21), including cafeterias, vending, and snack bars. Promote policies and programs that expand access to healthy food and beverages in community settings, including food retails, farmers markets, and restaurants (20).	Promote a brief dietary assessment as part of annual examination. Promote screening to measure the body mass index of patients during medical visits (www.uspreventiveservicestaskforce.org/Page/Document/UpdateSummaryFinal/obesity-in-adults-screening-and-management).	Promote physical activity, nutrition, and weight loss in chronic disease self-management programs.
Sleep Health–4: Increase the proportion of adults who get sufficient sleep	Conduct routine surveillance in BRFSS (adults) and YRBSS (adolescents). Conduct routine surveillance of school start times in school health profiles.	Promote later school start policies for adolescents (25–27). Promote healthy work shift schedules (27,28).	Promote sleep health screening and referrals to sleep specialists during medical visits (STOP–BANG^a^) (27,29).	Promote sleep health awareness in chronic disease self–management programs.

Abbreviations: BRFSS, Behavioral Risk Factor Surveillance System; YRBSS, Youth Risk Behavior Surveillance System; NYTS, National Youth Tobacco Survey; NATS, National Adult Tobacco Survey; YTS, Youth Tobacco Survey; SHPPS, School Health Policies and Practices Study.

a STOP–BANG sleep apnea questionnaire. STOP includes the following questions: 1) Do you SNORE loudly? 2) Do you often feel TIRED, fatigued, or sleepy during daytime? 3) Has anyone OBSERVED you stop breathing during your sleep? 4) Do you have or are you being treated for high blood PRESSURE? BANG includes the following questions: 1) BMI more than 35 kg/m^2^? 2) AGE over 50 years old? 3) NECK circumference greater than 16 inches (40 cm)? 4) GENDER is male?

Additional efforts are needed to increase the proportion of the population who engage in all 5 health-related behaviors addressed in this study. Collaborative efforts among public health agencies, health care systems, community coalitions, work sites, early child care and education, and schools can provide opportunities and support policies that promote these 5 behaviors and create population-wide changes, especially changes in racial/ethnic minority populations and socioeconomically disadvantaged populations. Coordinated, comprehensive approaches in clinical and public health sectors that address multiple behaviors or risk factors may allow more efficient leveraging of resources and increase the impact of interventions.
